# Sudden thrombus formation was detected during real-time ultrasound-guided central venous catheter insertion

**DOI:** 10.1186/s40981-025-00808-6

**Published:** 2025-08-22

**Authors:** Keita Uchiyama, Tsunehisa Tsubokawa

**Affiliations:** Department of Anesthesiology, The Jikei University School of Medicine, 3-25-8 Nishishimbashi, Minato-Ku, Tokyo, 105-8461 Japan

To the Editor

Catheter-related thrombosis occurs in 14–18% of the cases, typically several days after insertion and often following long-term use [1, 2]. We report a case of sudden thrombus formation, visualized in real time during catheter insertion under ultrasound guidance.

A 50-year-old man with dilated cardiomyopathy was admitted for worsening dyspnea. Transthoracic echocardiography revealed global left ventricular hypokinesis with a mobile apical thrombus (19 × 14 mm); the ejection fraction (EF) was 17%. Brain magnetic resonance imaging revealed multiple scattered cerebral infarctions, suggestive of embolic events. Surgical thrombectomy was scheduled. The patient had no history of previous surgery or central venous catheterization. Preoperative laboratory testing revealed a hemoglobin level of 15.1 g/dL, fibrinogen concentration 429 mg/dL, and platelet count of 240,000/mm^3^. After anesthesia induction, a central venous catheter was inserted into the right internal jugular vein under ultrasound guidance. No thrombus was visible during pre-scanning or guidewire insertion. However, upon insertion of the dilator, a thrombus suddenly appeared along the vascular wall (Supplementary video S1). The guidewire and dilator used in this procedure (MAC™ Two-Lumen Central Venous Access for use with 7.5–8 Fr. Catheters introducers, MTO-11142-AJ; Teleflex, USA) were not heparin-coated. The central venous catheter was inserted cautiously to prevent thrombus detachment. In this case, we prioritized immediate surgery over removal of the thrombus from the right internal jugular vein, owing to its relatively small size (8 × 12 mm). The thrombus was expected to be captured and removed by the cardiopulmonary bypass filter if dislodged and possibly dissolved by systemic heparinization. Following surgery, no symptoms or signs of a new cerebral infarction were observed.

To our knowledge, this is the first reported case in which acute thrombus formation was directly visualized during central venous catheter insertion. Although catheter-related thrombosis is commonly observed in cases of long-term catheter use, this case highlights the potential for immediate thrombus formation during the insertion process.

We hypothesize that thrombus formation in this case resulted from a combination of factors. First, ultrasound video revealed extravascular tissue being pulled into the vessel during dilator insertion, potentially exposing circulating blood to tissue factor (the image at 32 s and diagram in the supplement video). Second, the patient’s severely reduced EF may have caused venous stasis. EF reduction is a known independent risk factor for thromboembolic events [3]. Third, patients with dilated cardiomyopathy or low EF often show elevated coagulation markers [4], suggesting a hypercoagulable state. The interaction of these factors may have facilitated the immediate thrombus formation observed during dilator advancement.

While thrombotic risk factors were investigated post-surgery in this case, protein C and protein S activities, along with lupus anticoagulant levels, were within normal limits. Additionally, the patient had no family history of thrombotic disorders, and his clinical course ruled out heparin-induced thrombocytopenia as well.

This case underscores the importance of real-time ultrasound monitoring throughout the entire catheterization procedure. Continuous visualization under ultrasound guidance allows for immediate recognition of such thrombotic events and may help mitigate the risk of embolic complications through timely decision-making.

## Supplementary Information

Supplementary Material 1: video S1. Sudden thrombus formation during central venous catheter insertion. Real-time ultrasound recording showing sudden thrombus formation along the vascular wall during insertion of the dilator into the right internal jugular vein. No thrombus was observed during pre-scan or guidewire advancement. The thrombus developed abruptly upon insertion of the dilator. The guidewire and dilator used in this procedure were not heparin-coated.

## Data Availability

Not applicable.

## Abbreviation

EF: Ejection fraction
